# 22. THE NEAR FUTURE FOR SCHIZOPHRENIA (PSYCHOSIS) RESEARCH

**DOI:** 10.1093/schbul/sby014.089

**Published:** 2018-04-01

**Authors:** William T Carpenter

**Affiliations:** University of Maryland School of Medicine

## Abstract

**Overall Abstract:** Implications of a heterogeneous clinical syndrome such as schizophrenia have long been known but little attended. Fundamental problems persist, such as schizophrenia as the phenotype in GWAS studies. But the 21^st^ Century has brought substantial attention to limitations in acquisition of new knowledge. New concepts and methods are being implemented. Selected examples will be reviewed and potential scientific advances that influence clinical care will be outlined. This will include advances in mechanism knowledge, identification of novel targets for therapeutic discovery, re-conceptualization of psychopathology for regulatory purposes, a new integration of behavioral and biological science to inform nosology, enhanced testing of neural circuit hypotheses, and serious attention to primary prevention.

